# Supplementary material for: Phosphorylated Metal–Organic Framework for Reducing Fire Hazards of Poly(Methyl Methacrylate)
Source: Polymers (Basel). 2022 Nov 11;14(22):4871. doi: 10.3390/polym14224871 (PMC9697107; doi:10.3390/polym14224871)
Supplement: Supplementary file 1 [file polymers-14-04871-s001.zip › polymers-2004558-supplementary.pdf]

polymers

Supplementary Materials

Phosphorylated Metal–Organic Framework for Reducing Fire Hazards of Poly(Methyl Methacrylate)

Lei Wang[a], Ximiao Hu[a], Zhelin Mao[a], Jianlei Wang[b,*] and Xin Wang[a,]**

[a] College of Chemical Engineering and Material Science, Zhejiang University of Technology, Hangzhou, Zhejiang, 310014, China.
[b] Key Laboratory of Design and Assembly of Functional Nanostructures, Fujian Institute of Research on the Structure of Matter, Chinese Academy of Sciences, Fuzhou, Fujian, 350108, China

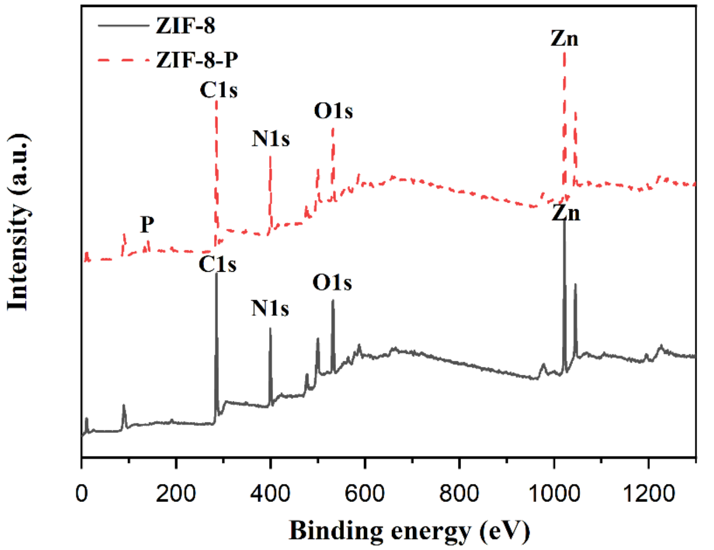

Figure S1. The XPS survey pattern of ZIF-8 and ZIF-8-P.

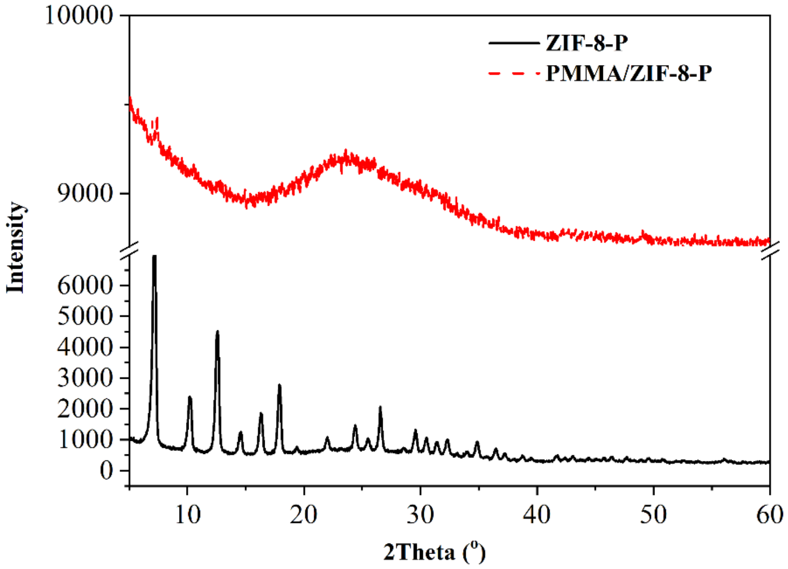

Figure S2. The XRD pattern of ZIF-8-P and PMMA/ZIF-8-P composite.
